# Clinical Lectures on the Diseases of Women and Children

**Published:** 1860-05

**Authors:** 


ART. IX.—*Clinical Lectures on the Diseases of Women and Children.* By GUNNING S. BEDFORD, A.M., M.D., Professor of Obstetrics, the Diseases of Women and Children, and of Clinical Midwifery in the University of New York. Sixth edition. 8vo., pp. 653. New York: Samuel S. & William Wood, 1860.

THE success of this work must be alike gratifying to its distinguished author and to his enterprising publishers. No medical publication has ever appeared in this country which has received higher or more flattering encomiums from the periodical press at home and abroad. The *London Medical Times and Gazette, London Lancet, British and Foreign Medico-Chirurgical Review, British Medical Journal,* and *Gazette Médicale de Paris,* all speak of it as a work of great practical value, and of the author as a man of unusual talent for observation. The fact that it has passed to a sixth edition since its first appearance in July, 1855, is a sufficient evidence of the estimation in which it is held by the American profession. Two additional lectures have been incorporated in the present edition, thereby considerably enhancing its value.

If we felt disposed to exercise our rights as critics, it would be easy enough to point out a number of objectionable features in the work before us. Its faults, indeed, are not a few. The style in which it is written is, in particular, distasteful; and as to the arrangement of its topics, hardly anything could be more irregular and disorderly. As a nosographer, Dr. Bedford evidently possesses talents of a high order, and there are few practitioners who would not feel disposed to indorse, in the main, his practical precepts. Indeed, the whole work is eminently practical, and this circumstance is, we doubt not, the great secret of its extraordinary success.

